# Hepatitis E virus RNA replication polyprotein: taking structural biology seriously

**DOI:** 10.3389/fmicb.2023.1254741

**Published:** 2023-08-04

**Authors:** Sonia Fieulaine, Thibault Tubiana, Stéphane Bressanelli

**Affiliations:** Université Paris-Saclay, CEA, CNRS, Institute for Integrative Biology of the Cell (I2BC), Gif-sur-Yvette, France

**Keywords:** hepatitis E virus (HEV), replication polyprotein pORF1, structural biology, AlphaFold2, bioinformatics

Hepatitis E virus (HEV) infects approximately 20 million individuals each year all around the world, both in developing and industrialized countries. It leads to 40,000–70,000 deaths annually, especially in immunocompromised patients and pregnant women. Despite its recognized major public health issue status and zoonotic potential, no specific treatment is available. Indeed, HEV life cycle characterization is hampered by the lack of efficient infectious cell culture systems or *in vivo* models. A better knowledge of HEV virology is therefore needed. By providing descriptions of the three-dimensional structures of viral proteins at atomic level, structural biology can be a powerful tool to understand viral replication and help develop specific antivirals. In this comment, we describe how both experimental and advanced computational structural biology help to decipher HEV virology and make a case for heeding its lessons.

HEV pORF1 is the replication polyprotein encoded by open reading frame 1. It contains domains that ensure the synthesis of new viral RNA genomes in infected cells. As for other single-stranded positive-sense RNA viruses [(+)RNA viruses], it encodes the viral replication complex (prominently, the RNA-polymerase) and presumably includes domain(s) that allow targeting to and remodeling of a specific host endomembrane to shelter the replication complex. pORF1 organization was delineated promptly after virus identification in the early 1980's (Khuroo, 1980; Balayan et al., 1983) and genome sequencing in the 1990's (Reyes et al., 1990; Tam et al., 1991). In 1992, Koonin *et al*. used sequence-based computational tools to perform sequence alignments with some closely related (+)RNA viruses belonging to the Alphavirus-like superfamily and define domain boundaries of pORF1 (Koonin et al., 1992). They tentatively proposed six domains embedded in pORF1. In decreasing order of confidence, these are the aforementioned RNA-dependent RNA-polymerase (RdRp, residues 1,207–1,693 for the genotype 1 (gt1) strain they analyzed), an RNA helicase (HEL, residues 960–1,192), a methyltransferase (Met, residues 56–240), a Y domain (residues 219–433), an X domain (residues 784–942) and a papain-like cysteine protease (PCP, residues 434–592). A proline-rich hypervariable region is sometimes considered as a 7^th^ domain. As mentioned by the authors then, the confidence index for the putative PCP domain prediction was very low and they proposed a protease in HEV pORF1 mainly because it was already known in other animal (+)RNA viruses. Since then, the HEV community has taken to referring to HEV pORF1 residues 434–592 as “the PCP domain” or “the HEV protease.”

However, structural biology has recently shown that this initial assignment was erroneous, both with an experimental X-ray crystal structure and with groundbreaking computational tools, including those based on artificial intelligence (AI). As summarized below, it is now clear that HEV pORF1 does not contain any protease domain, either in residues 434–592 or anywhere else.

First, in 2019 Proudfoot et al. solved the crystal structure of the 510–690 fragment of a gt1 HEV pORF1 (Proudfoot et al., 2019). Structurally, this fragment is unequivocally a member of a large protein family known as fatty acid binding proteins. Even though the role of this HEV fatty acid binding domain (FABD)-like domain during the viral life cycle is not yet known, it fits the definition of a protein domain, *i.e*. a region of a protein that is self-stabilizing and folds, functions and evolves independently from the rest. Thus, it is actually established since 2019 that residues 434–592 cannot be a protease domain.

In recent years, the AI-based AlphaFold2 (AF2) tool (Jumper et al., 2021) revolutionized the field of sequence-based protein structure prediction, providing, from protein sequences alone, structural models with high accuracy and very good estimation of the error in the coordinates (Jumper et al., 2021; Tunyasuvunakool et al., 2021). Three groups, including ours, have used AF2 to generate accurate structural models of HEV pORF1, either in its full-length form (Fieulaine et al., 2023; LeDesma et al., 2023) or segmented into two overlapping fragments [1–1,250 and 1,000–1,708 (Goulet et al., 2022)]. All the three groups independently obtained similar models, exhibiting five domains with very high confidence scores, and a long disordered region corresponding to the hypervariable, proline-rich region (Figure 1A). Upstream this region, the models fuse the proposed Met and Y domains into a single domain MetY clearly homologous to the Alphavirus nsP1, and find the aforementioned FABD-like domain, perfectly superimposable to the crystal structure. Taken together, experimental and computational structural data leave no place for a domain in position 434–592 and thus demonstrate that there is no PCP in HEV pORF1. An intriguing possibility is that pORF1 functions could be regulated not by proteolytic processing but rather by structural flexibility of different motifs: Indeed both the N- and C-terminal α-helices of MetY seem to alternate between unfolded and folded states, the C-terminal extension of the FABD-like domain could open to allow the binding of yet unidentified ligand(s), the RdRp could alternate between different conformations especially in its fingertips motif (Fieulaine et al., 2023). In this respect, decade-long studies on the distantly related flock house virus, whose counterpart to HEV pORF1 is not cleaved, has recently culminated in remarkable structural and cellular work (Zhan et al., 2023). This latter work establishes how flexible linkers allowing large conformational switches can be used to build a replication complex harboring all major functions for (+)RNA virus replication through formation of a large oligomeric ring of the uncleaved replication polyprotein.

**Figure 1:**
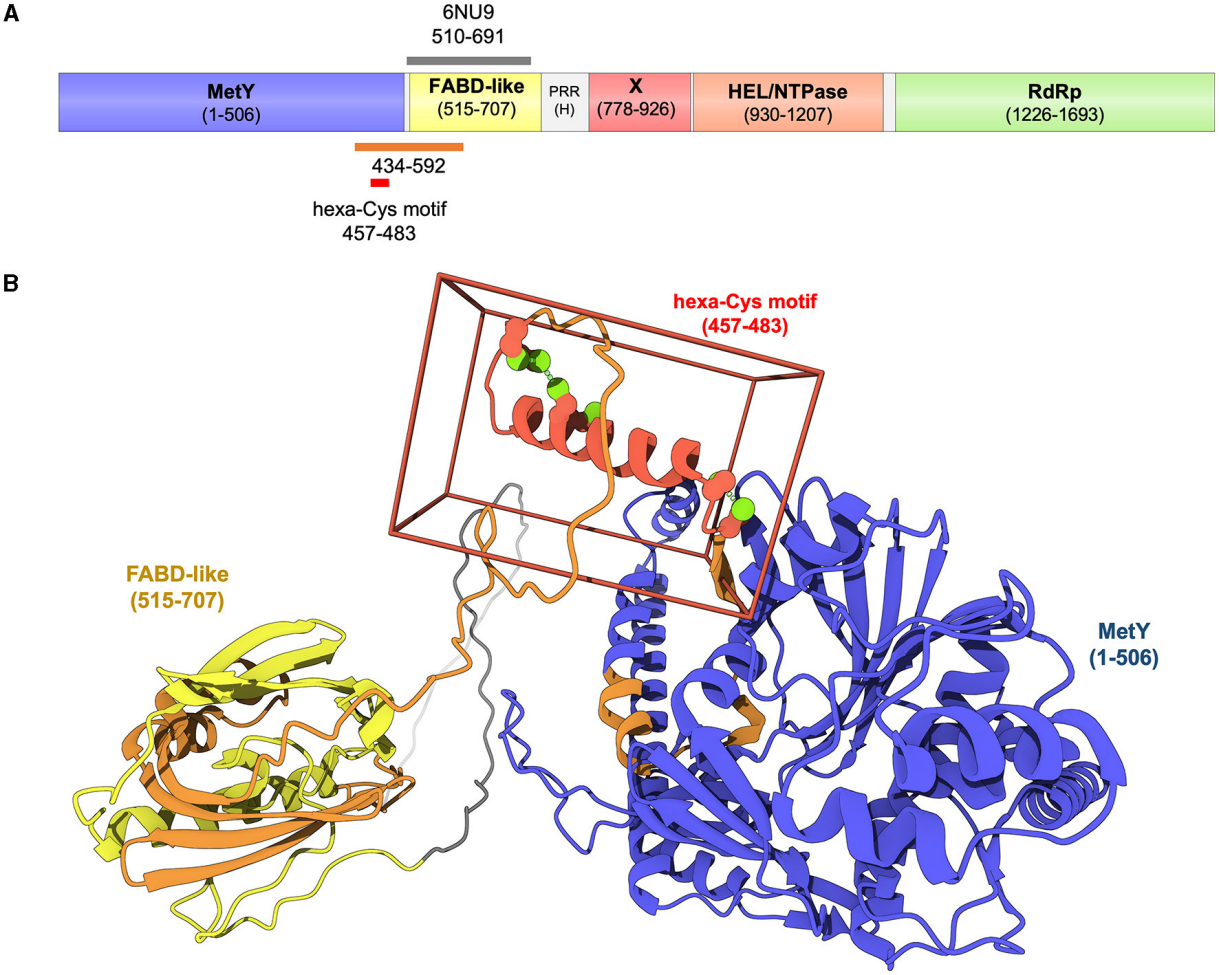
Domain organization of genotype 1 HEV pORF1 as delineated by experimental and computational structural biology (Proudfoot et al., 2019; Goulet et al., 2022; Fieulaine et al., 2023; LeDesma et al., 2023). **(A)** HEV pORF1 contains five independent domains: From N-terminus to C-terminus a methyltransferase-and-membrane-binding domain (MetY in blue), a FABD-like domain (in yellow), a macrodomain-X (in red), a helicase [HEL/NTPase in orange) domain and an RNA-dependent RNA polymerase (RdRp) domain (in green). A proline-rich (PRR) hypervariable region (H, in gray)] is found between the N-terminal module (MetY-FABD) and the C-terminal module (X-HEL-RdRp). Orange bar, region 434–592 initially and tentatively assigned to a protease domain (Koonin et al., 1992). Gray bar, 510–691 crystal structure with PDB ID 6NU9 (Proudfoot et al., 2019). Red bar, hexa-Cys motif extensively mutated by LeDesma et al. (2023). **(B)** Structure of the N-terminal module of HEV pORF1 as modeled with AlphaFold2 (Fieulaine et al., 2023). Cartoon representation including MetY and FABD-like domains as well as specific regions colored as in **(A)**.

We are at a watershed in HEV biology in which too many researchers still refer to the HEV PCP, despite the hard data we just outlined. This situation is very well exemplified by the work of LeDesma et al. recently published in eLife (LeDesma et al., 2023). These authors set out to probe the function of the putative PCP and also reached the conclusion that it is not a protease, that pORF1 is likely not cleaved, and that regulating pORF1 structure is crucial for its functions (Dearborn et al., 2023). Importantly, several cysteines, including Cys483 that was initially proposed to be the catalytic cysteine of the HEV protease, do play a role during viral replication. These Cys are found in a hexa-Cys motif (CxCx_11_CCx_8_CxC in region 457–483) that is likely to bind divalent cations, most probably zinc (Dearborn et al., 2023; LeDesma et al., 2023). Strikingly, this motif corresponds to the 461–477 α-helix located at the C-terminus of the MetY domain (Figure 1B) that we proposed would play a central role during MetY oligomerization and binding to yet unidentified membranes (Fieulaine et al., 2023).

In the light on these new data, we think it is time to stop using the terms “HEV protease” or “PCP domain,” as the corresponding region is now established to be neither a PCP nor even a domain, and to heed the contribution of structural biology in probing the actual functions of this part of HEV pORF1.

## Author contributions

SF: Conceptualization, Funding acquisition, Writing—original draft, Writing—review and editing. TT: Visualization, Writing—review and editing. SB: Conceptualization, Funding acquisition, Writing—review and editing.

## References

[B1] Balayan M. S. Andjaparidze A. G. Savinskaya S. S. Ketiladze E. S. Braginsky D. M. Savinov A. P.. (1983). Evidence for a virus in non-A, non-B hepatitis transmitted via the fecal-oral route. Intervirology. 20, 23–31. 10.1159/000149370 6409836

[B2] Dearborn A. D. Kumar A. Marcotrigiano J. (2023). Learning more about hepatitis E virus. eLife. 12, e87047. 10.7554/eLife.87047 36947136 PMC10032651

[B3] Fieulaine S. Tubiana T. Bressanelli S. (2023). De novo modelling of HEV replication polyprotein: five-domain breakdown and involvement of flexibility in functional regulation. Virology. 578, 128–140. 10.1016/j.virol.2022.12.002 36527931

[B4] Goulet A. Cambillau C. Roussel A. Imbert I. (2022). Structure prediction and analysis of hepatitis E virus non-structural proteins from the replication and transcription machinery by AlphaFold2. Viruses. 14, 1537. 10.3390/v14071537 35891516 PMC9316534

[B5] Jumper J. Evans R. Pritzel A. Green T. Figurnov M. Ronneberger O.. (2021). Highly accurate protein structure prediction with AlphaFold. Nature. 596, 583–589. 10.1038/s41586-021-03819-2 34265844 PMC8371605

[B6] Khuroo M. S. (1980). Study of an epidemic of non-A, non-B hepatitis: possibility of another human hepatitis virus distinct from post-transfusion non-A, non-B type. Am. J. Med. 68, 818–824. 10.1016/0002-9343(80)90200-4 6770682

[B7] Koonin E. V. Gorbalenya A. E. Purdy M. A. Rozanov M. N. Reyes G. R. Bradley D. W.. (1992). Computer-assisted assignment of functional domains in the nonstructural polyprotein of hepatitis E virus: delineation of an additional group of positive-strand RNA plant and animal viruses. Proc. Natl. Acad. Sci. 89, 8259–8263. 10.1073/pnas.89.17.8259 1518855 PMC49897

[B8] LeDesma R. Heller B. Biswas A. Maya S. Gili S. Higgins J.. (2023). Structural features stabilized by divalent cation coordination within hepatitis E virus ORF1 are critical for viral replication. eLife. 12, e80529. 10.7554/eLife.80529.sa2 36852909 PMC9977285

[B9] Proudfoot A. Hyrina A. Holdorf M. Frank A. O. Bussiere D. (2019). First crystal structure of a nonstructural hepatitis E viral protein identifies a putative novel zinc-binding protein. J. Virol. 93, 10–1128. 10.1128/JVI.00170-19 31019049 PMC6580954

[B10] Reyes G. R. Purdy M. A. Kim J. P. Luk K. C. Young L. M. Fry K. E.. (1990). Isolation of a cDNA from the virus responsible for enterically transmitted non-A, non-B hepatitis. Science. 247, 1335–1339. 10.1126/science.2107574 2107574

[B11] Tam A. W. Smith M. M. Guerra M. E. Huang C-. C. Bradley D. W.. (1991). Hepatitis E virus (HEV): molecular cloning and sequencing of the full-length viral genome. Virology. 185, 120–131. 10.1016/0042-6822(91)90760-9 1926770 PMC7130833

[B12] Tunyasuvunakool K. Adler J. Wu Z. Green T. Zielinski M. Žídek A.. (2021). Highly accurate protein structure prediction for the human proteome. Nature. 596, 590–596. 10.1038/s41586-021-03828-1 34293799 PMC8387240

[B13] Zhan H. Unchwaniwala N. Rebolledo-Viveros A. Pennington J. Horswill M. Broadberry R.. (2023). Nodavirus RNA replication crown architecture reveals proto-crown precursor and viral protein A conformational switching. Proc. Natl. Acad. Sci. U S A. 120, e2217412120. 10.1073/pnas.2217412120 36693094 PMC9945985

